# Prevalence of bovine brucellosis in slaughtered cattle and barriers to better protection of abattoir workers in Ibadan, South-Western Nigeria

**DOI:** 10.11604/pamj.2017.28.68.10925

**Published:** 2017-09-22

**Authors:** Modupe Comfort Ayoola, Victor Oluwatoyin Akinseye, Eniola Cadmus, Emmanuel Awosanya, Olufemi Akinyele Popoola, Oluwaseun Oladapo Akinyemi, Lorraine Perrett, Andrew Taylor, Judy Stack, Ignacio Moriyon, Simeon Idowu Cadmus

**Affiliations:** 1 Department of Veterinary Public Health and Preventive Medicine, Faculty of Veterinary Medicine, University of Ibadan, Ibadan, Nigeria; 2 Department of Community Medicine, Faculty of Clinical Sciences, College of Medicine, University of Ibadan, Ibadan, Nigeria; 3 Department of Health Policy and Management, Faculty of Public Health, College of Medicine, University of Ibadan, Ibadan, Nigeria; 4 Department of Bacteriology, Animal and Plant Health Agency, Surrey, United Kingdom; 5 Instituto de Salud Tropical y Departamento, Microbiología y Parasitología, Universidad de Navarra, Edificio de Investigación, c/Irunlarrea 1, 31008 Pamplona, Spain

**Keywords:** Brucellosis, prevalence, slaughtered cattle, protective wears, abattoir workers

## Abstract

**Introduction:**

Brucellosis is a neglected zoonosis of public health importance. This study was conducted to determine the prevalence and risk factors of brucellosis among slaughtered cattle as well as challenges to the protection of abattoir workers in Nigeria.

**Methods:**

A slaughterhouse study was conducted in a major abattoir in Ibadan from March to August, 2013. To diagnose brucellosis, serum samples from 1,241 slaughtered cattle were tested using Rose-Bengal test (RBT) and competitive enzyme-linked immunosorbent assay (cELISA); again, 57 milk samples were tested with milk ring test (MRT) and indirect ELISA (iELISA). Furthermore, a survey on the usage of personal protective equipment (PPE) and challenges to its use by abattoir workers was done. Data were analysed using Stata 12.

**Results:**

Seroprevalence by RBT was 7.8%; 77.3% (75/97) of these were corroborated by cELISA. Prevalence in milk samples by MRT and indirect ELISA were 33.3% and 3.5%, respectively. Sex (OR: 2.5; 95%CI:1.3-4.5) was the factor significantly associated with *Brucella* seropositivity. None of the abattoir workers used standard protective overalls; while, 99.6% of the meat handlers and 84.1% of the butchers worked barefoot. Most of the workers (75.7%) wore no protective gloves. The respondents agreed that provision of free PPE and sanctions against non-users would encourage its use.

**Conclusion:**

Our findings indicate moderate prevalence (7.8%) of bovine brucellosis with sex of cattle being a risk factor. A notable barrier to better protection of abattoir workers against brucellosis is perceived inconvenience arising from use of gloves. Therefore, preventive and control measures against brucellosis must include education and use of PPE among abattoir workers.

## Introduction

Brucellosis is one of the most virulent infectious and zoonotic diseases of global public health relevance [1]. The disease affects a wide range of animals and it is caused by organisms belonging to the genus *Brucella* including species like *B. melitensis* (in sheep and goats), *B. abortus* (in cattle), *B. suis* (in pigs), *B. canis* (in dogs), *B. ovis* (in sheep) and *B. neotame* (in wood rats). In animals and humans, it leads to reproductive and diverse clinico-pathological sequelae. Infections in bulls cause orchitis, epididymitis, seminal vesiculitis and hygroma [2]. In cows, it results in abortions, stillbirth, birth of weak calves and reduced milk production. Humans present clinical manifestations including undulant fever, weakness, orchitis and, if left untreated, it can result in osteomyelitis, arthritis, meningitis and endocarditis [3], which may lead to painful death [4] in 1-5% of the cases [5]. The most dependable method of diagnosing brucellosis is isolation of the causal agent [6, 7]. However, serological methods have proven useful in the study of brucellosis in developing countries because they are simple, cost effective, robust and reproducible [8, 9]. Various serological tests have been deployed for screening of brucellosis in humans and animals; of these, the RBT has been recommended, particularly in areas where vaccination of animals is not practised, due to its high sensitivity and simple technique as well as a relatively low cost [10]. Nevertheless, since the World Organisation for Animal Health (OIE) recommends at least two serological tests [11], the competitive ELISA (c-ELISA) and indirect ELISA (iELISA) which have proved useful in several previous studies, were used as confirmatory tests to the RBT and the milk ring test (MRT) [12–15]. Though primarily a disease of animals, brucellosis also affects humans; but, with more profound impact in developing countries where public and animal health programmes are weak [16]. Hence, brucellosis is considered a neglected zoonosis in most African countries including Nigeria, due to limited efforts directed at its control [17,18] and precisely because of the category of people affected (particularly the poorly educated).

Among those infected, contact with contaminated substances of animal origin as well as inhalation of aerosolised organism are the primary causes [19]. Human brucellosis, is therefore a zoonotic and an important occupational disease [11] that is common among livestock workers and veterinarians [20–22]. However, livestock workers are more prone to infections because of their frequent exposure to blood, discharges, carcasses and viscera of infected animals through cuts and wounds [23], as well as splashes from infected blood into their conjunctiva and inhalation of aerosols [23, 24]. Abattoir and livestock workers in Nigeria have a high risk of exposure to brucellosis due to poor hygiene, poor workplace safety standards as well as ignorance of the disease [25]. Despite these obvious risks and exposure potentials, few studies have explored livestock workers behavioural tendencies towards contracting brucellosis in Nigeria (a country with the highest human population in Africa) [26, 27]. This gap in knowledge, therefore, has a major impact on our understanding of the risk behaviours related to transmission of brucellosis among livestock workers. In general, abattoir workers are likely to take health-related precautionary measures to protect themselves (for example by using personal protective equipment (PPE) during procedures that could predispose to brucellosis. Hence, we set out to determine the prevalence of bovine brucellosis and associated risk factors among slaughtered cattle in Ibadan, Nigeria against the backdrop of examining work related risk behaviours and barriers to effective protection of abattoir workers from *Brucella* infection.

## Methods

### Study setting

**Figure 1:**
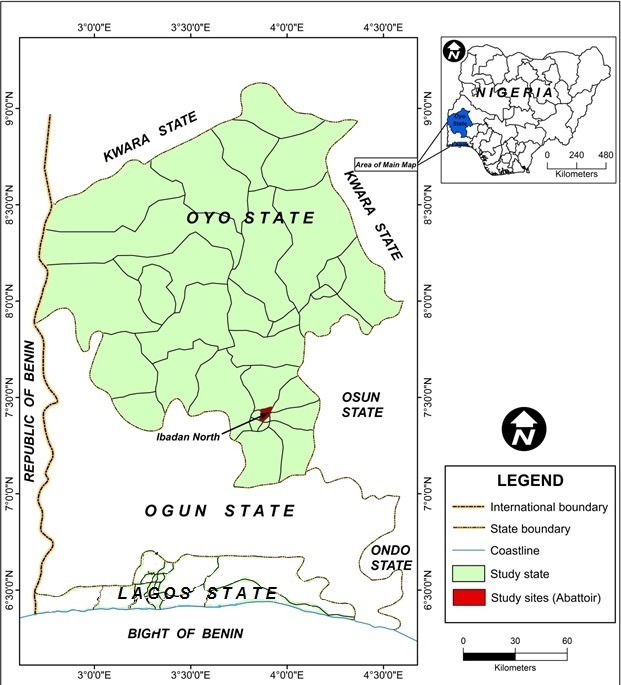
Map of Oyo State showing Ibadan north, the study site (map of Nigeria inset).

This study was conducted at Bodija Municipal Abattoir, located in Ibadan, Oyo State, South-western Nigeria (Figure 1). Ibadan is the largest city in West Africa with over 1.3 million inhabitants [28] and the third largest in Africa, after Cairo and Johannesburg. The city is a prominent transit point between the coastal region and northern part of Nigeria. Bodija Municipal Abattoir, though the main facility for slaughtering livestock animals in Ibadan, is plagued by poor sanitary and hygiene standards. Cattle slaughtered at the abattoir are sourced from Akinyele International Cattle Market, a major cattle market in the state that receives its cattle mass from the northern part of Nigeria and neighbouring African countries like Burkina Faso, Cameroon, Chad, Mali and Niger (Figure 1).

### Study design and sample size

A slaughterhouse study was carried out from March to August, 2013. A systematic random sampling technique was used for animal selection. On an average, 200-250 cattle were slaughtered on daily basis. Based on the calculated sampling fraction of five, every fifth animal was sampled. Daily, the first animal was selected by balloting between 1 to 5. Thereafter, every fifth animal (by adding 5 to the initial number picked) was selected till the sample size was achieved. To calculate the required sample size, we adopted the cross sectional formula using a prevalence of 39% [29]:

$$n=\frac{1.96^{2}.p.(1-p)}{d^{2}}$$

Using this formula, we arrived at a sample size of 303. However, in order to accommodate a wider range of samples, we collected 1241 samples. By our estimation, there were approximately 350 workers in the abattoir during the study. A structured (questions with specified options) questionnaire was given to all consenting abattoir workers. A total of 239 respondents consented to be interviewed.

### Sample collection and storage

About 5ml of blood was collected from the jugular vein of selected cattle, immediately after slaughter, into a sterile vacutainer tube. The blood samples were slanted, allowed to clot and kept in cool containers. The samples were transported in flasks with ice packs to the Tuberculosis and Brucellosis Laboratories, Department of Veterinary Public Health and Preventive Medicine, University of Ibadan for processing. Blood samples were centrifuged after 24 hours at 3000g for five minutes (800rev/min) and serum were thereafter decanted and stored at -20^o^C until they were assayed. Milk samples (10ml) were also collected from udders of lactating cows, not showing clinical mastitis, into 20ml sterile milk sample bottles.

### Tests and assays

Serum samples were tested by RBT using equal volume of the serum and the RBT antigen as earlier described (6) and positive samples were subjected to c-ELISA according to Stack et al. (1999) [30]. Milk samples were examined using the MRT and i-ELISA according to the OIE stipulations [31]. Samples were classified as positive or negative as recommended by the manufacturer’s cut-off ranges. All test reagents and kits were obtained from Animal and Plant Health Agency (APHA), Surrey KT15 3NB, United Kingdom.

### Daily observations

In this study, we identified the personal protective equipment (PPE) such as overall/coat, rubber gloves, rubber or plastic apron, face mask, boots and eye shield that were worn by abattoir workers to prevent risks of infection at their workplace. Evaluation of workers’ risk behaviours were carried out through daily observations of the use of PPE. Records were kept on the protective clothing worn by each abattoir worker while processing cattle carcass, as well as the number of pregnant women and children (women and children were also sometimes involved in meat/offal processing activities within the slaughter halls) at the abattoir each day throughout the duration of study. In addition to the aforementioned (PPE observed), the breed, sex, age and body score condition of the animals were recorded. Based on modification of body condition score by Moran (2005) [32], the body condition score of animals in this study were categorised into four groups: i) Good - cattle with no prominent bone appearing through the skin and showing evidence of fat deposit; ii) Moderate - cattle with no prominent bone appearing through the skin and with little or no evidence of fat deposit; iii) Emaciated – thin cattle with prominent ribs and hip bones; iv) Highly emaciated – cattle with very thin covering of flesh, sharply protruding ribs and hip bones with great depression of the lumbar fossa.

### Questionnaire

Structured (questions with specified options) and pretested questionnaire was used to obtain data on socio-demographic characteristics of respondents, their perceived barriers to usage of PPE, self-efficacy and willingness to action towards better protection. The questionnaire was completed by interview with the workers. The questionnaire comprised of 20 questions. Respondents were asked to identify their level of agreement using the 5-point Likert scale (strongly agree, agree, don’t know, disagree, strongly disagree).

### Data analysis

Data were analysed using Stata version 12. Frequencies were generated and fisher exact test was used to explore variables potentially associated with *Brucella* infection among cattle. The level of statistical significance was set at p < 0.05.

### Ethical approval

Ethical approval for this work was obtained from University of Ibadan/University College Hospital Institution Ethical Review Board (NHREC/05/01/2008a).

## Results

### Serological analysis

A total of 1241 animals were sampled of which 97 (7.8%) were seropositive to *Brucella* infection. As shown in Table 1, among the seropositive animals, 59.8% were of the Bunaji breed while the lowest (3.1%) were of the Kuri breed. Also, more female cattle (85.6%) were seropositive to *Brucella* antibodies than males; while emaciated cattle, recorded the highest percentage (68.0%); as those with good, moderate and highly emaciated body scores having 25.8%, 1.0%, and 5.2% respectively among the seropositive animals. All infected adult cattle were above 3 years of age. Univariate analysis revealed a statistically significant association between sex of the animals in relation to animal seropositivity (p < 0.001); while the breed ((p = 0.50) and body condition (p = 0.88) of animals were however not significantly associated with it (Table 1).

**Table 1:** Univariate analysis of intrinsic factors associated with Brucella seropositivity by Rose Bengal test in Ibadan South-western Nigeria

Variables	Category	Brucella seropositivity by RBT		OR	95%CI	P-value
		Positive n (%)	Negative n (%)			
Breed	Bunaji	58 (59.8)	632 (55.2)	1		
	Rahaji	20 (20.6)	270 (23.6)	0.8	0.5 – 1.4	0.50
	Sokoto Gudali	7 (7.2)	64 (5.6)	1.2	0.5 – 2.7	0.84
	Mixed breed	9 (9.3)	161 (14.1)	0.6	0.3 – 1.3	0.23
	Kuri	3 (3.1)	17 (1.5)	1.9	0.5 – 6.8	0.53
Sex	Male	14 (14.4)	339 (29.6)	1		
	Female	83 (85.6)	805 (70.4)	2.5	1.3 – 4.5	0.002
Body score	Good	25 (25.8)	365 (31.9)	1.7	0.2- 13.7	0.88
	Moderate	1 (1.0)	26 (2.3)	1		
	Emaciated	66 (68.0)	685 (59.9)	2.5	0.3 – 18.8	
	Highly emaciated	5 (5.2)	68 (5.9)	1.9	0.2 – 17.2	0.91
Age	Adult	97 (100.0)	1141 (99.7)	---	----	
	Young	0 (0.0)	3 (0.3)	---	----	

### MRT and i-ELISA

A total of 57 milk samples were collected at the abattoir of which 33.3% were positive by MRT while 3.5% were positive by i-ELISA.

### Daily observation

**Figure 2:**
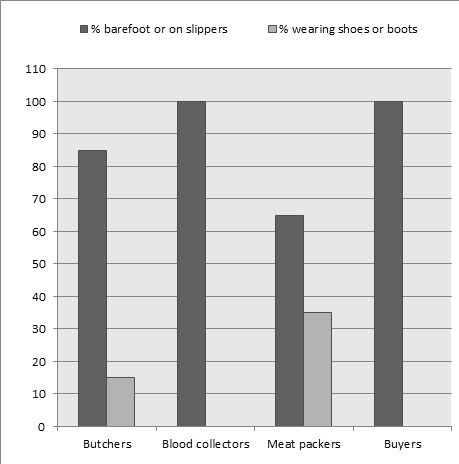
Daily observation on the use of personal protective equipment (PPE) among livestock workers.

Result of daily observation regarding the use of PPE by the abattoir workers showed that none of them used any protective clothing. However, they were in the habit of putting on different clothing not designated for such purposes when processing meat, except for the veterinarians and para-veterinarians that used few of the PPE (overall, boots and gloves). Majority of the butchers observed were either bare-foot or wore slippers. Similarly, almost all the meat handlers wore slippers while the blood packers wore neither shoes nor boot (Figure 2). Finally, on a daily basis, an average of three pregnant women worked at the abattoir and three children were brought in by working mothers during slaughtering and meat processing (Figure 2) (*Footwear was the only PPE used by the livestock workers).

### Questionnaire

A total of 248 abattoir workers were approached to participate in the study out of which 239 agreed. Others declined because of their busy schedule as well as the need to get the processed meat to the market early enough. Most of the respondents (79.9%) were males, and more than three quarters were above 30 years (79.5%) and 60.3% had over 10 years work experience (Table 2). Almost all the respondents (95.4%) indicated that having open wound while processing meat will not stop them from working and believed that the cost was not a barrier against the use of PPE (99.6%). Majority (75.7%) did not use gloves because of a perceived inconvenience arising from its use (Table 3). Almost all the respondents (93.7%) strongly agreed that there was a need for an educational programme to create awareness about brucellosis and ways to prevent disease transmission. The most (94.6%) preferred medium of transmission was through radio programmes (Table 4). Most of the respondents (96.7%) agreed that provision of PPE free of charge will help protect against diseases in the abattoir. All the respondents agreed that introduction of penalties by the abattoir association leaders against defaulters would motivate beneficial behavioural change.

**Table 2:** Socio-demographic characteristics and work experience of respondents at Bodija abattoir, Ibadan South-western Nigeria

Variable	Frequency (n)	Percentage (%)
**Age (years)**		
<30	49	20.5
>30	190	79.5
**Sex**		
Male	191	79.9
Female	48	20.1
**Marital status**		
Single	16	6.7
Married	223	93.3
**Educational status**		
No formal education	37	15.5
Primary	117	48.9
Secondary	70	29.3
Tertiary	15	6.3
**Work category**		
Butchers and slaughterers	117	48.9
Cattle/meat handlers	110	46.0
Fetus processor	3	1.3
Meat packers/carriers	3	1.3
Vets/paravets	6	2.5
**Years of work experience**		
0-10	94	39.3
10 and above	145	60.7

**Table 3:** Perceived barriers to prevention of abattoir workers against brucellosis in Ibadan South-western Nigeria, 2013 (n = 239)

Perceived barriers to prevention n (%)	Strongly agree/agree	Strongly disagree/disagree
I can’t wear protective overall because it is not the custom where I work	28 (11.7)	211 (88.3)
I can’t wear protective overall because they are expensive	1 (0.4)	238 (99.6)
I don’t wear protective overall because my colleagues do not	4 (1.7)	235 (98.3)
I can’t wear boots because it is not the custom where I work	38 (15.9)	201 (84.1)
I can’t wear boots because they are expensive	12 (5.0)	227 (95.0)
I don’t wear boots because my colleagues do not	3 (1.3)	236 (98.7)
I can’t wear hand-gloves because it is not the custom where I work	39 (16.3)	200 (83.7)
I can’t wear hand-gloves because they are expensive	0 (0.0)	239 (100.0)
I don’t wear hand-gloves because my colleagues do not	3 (1.3)	236 (98.7)
I don’t wear hand-gloves because it is not convenient for my work	181(75.7)	58 (24.3)
I can’t wear face-mask because it is not the custom	17 (7.1)	222 (92.9)
Having a cut on my hand cannot stop me from handling meat	228 (95.4)	11 (4.6)

**Table 4:** Self-efficacy and cues to action towards protection of abattoir workers against brucellosis in Ibadan South-western Nigeria, 2013 (n = 239)

	Strongly agree/agree	Strongly disagree/disagree
**Self-efficacy n (%)**		
I can afford protective wears in order to protect myself	238 (99.6)	1 (0.4)
I can wear protective wear even if my colleagues do not	239 (100.0)	0 (0.0)
I am able to tell if carcasses may be infected	238 (99.6)	1 (0.4)
**Cues to action n (%)**		
I need educational programs to teach me how to protect myself against contracting brucellosis at my place of work.	224 (93.7)	15 (6.3)
I need free protective clothing to protect myself against contracting brucellosis at my place of work.	231 (96.7)	8 (3.4)
Imposed penalties by abattoir association leaders would enforce wearing protective clothing	239 (100.0)	0 (0.0)
Radio advertisements would encourage wearing of protective clothing at abattoir	226 (94.6)	13 (5.4)

## Discussion

Judging from the risks and exposure potentials of livestock workers to brucellosis, we examined work related risk behaviours and barriers to effective protection of abattoir workers against *Brucella* infection in a large slaughter house setting in Ibadan, southwestern Nigeria. Focussing on PPE, our findings reveal that a major barrier to its usage (particularly gloves) among abattoir workers was perceived inconvenience arising from its use. More so, we observed that majority of the respondents would not be deterred from handling meat and other animal products despite having cuts or wounds to their hands. The plausible reason for these risky behaviours was linked to ignorance or poor knowledge about the risk of exposure to zoonotic diseases which could be contracted through unguarded contact with blood, meat and other by-products during meat processing [25]. Though, most of the respondents posited that the cost of PPE was not a barrier to its usage; notably, majority (96.7%) agreed that provision of free PPE would serve as an inducement for its usage [22, 33]. This study also revealed respondents’ preference for prompts and reminders to take preventive actions against brucellosis, and these included enlightenment programmes on protection against brucellosis, imposed penalties by abattoir association leaders and radio advertisement. The enlightenment and education of abattoir workers about brucellosis could be done through organised educational programmes like seminars/workshops. Again, the media’s role in general health education targeting abattoir workers was highlighted. Since the magnitude of the cue required to trigger preventive and beneficial health actions would depend on the motivation to change and the perceived benefit to cost ratio for the action [34], free PPE and imposed penalties may trigger the workers to protect themselves against brucellosis. Hence, cues to action may serve as means of behavioural change as observed in this study.

Furthermore, we observed that most workers in the abattoir did not use standard protective overall but only wore casual clothing when processing carcasses. Majority of them do not use adequate protective footwear (boot) while working at the abattoir. This situation is similar to the findings of Swai and Schoonman (2009) [35] who noted that in Tanzania, livestock workers were at great risk of contracting brucellosis because of their practice of handling animal tissues without the use of protective gear. The inadequate use of PPE among these workers put them at grave risk of exposure to brucellosis and other zoonotic diseases, especially as they engaged in unhygienic practices within a heavily contaminated setting [36, 37]. However, we observed a higher adoption in the use of PPE among veterinarians and para-veterinarians than other workers such as the butchers and meat handlers. This also, could be adduced to the formal education and or better knowledge of brucellosis by veterinarians and para-veterinarians than other abattoir workers. Socio-demographic factors, particularly educational attainment, are believed to have an indirect effect on behaviour by influencing the perception of susceptibility to infectious diseases [38]. However, poor knowledge of the disease could potentially influence the transmission of infection, delay diagnosis and ultimately treatment [39]. This implies that individuals, who believe that they are at risk of a disease, will take healthy preventive measures through behavioural change [40]. However, those without its knowledge or implication of being infected will not protect themselves even though they may be at high risk. Again, given the unhygienic conditions at the abattoir, most pregnant women working there may be at high risk of exposure to *Brucella* infections. Since pregnancy increases susceptibility to generalised *Brucella* infection [41], therefore, brucellosis in pregnancy poses a substantial risk of spontaneous abortion [42]. Again, the presence of children and their being fed within such contaminated facility (sometimes with unwashed hands) may equally put them at the risk of exposure to Brucella infections. Importantly, in societies where brucellosis is endemic, it becomes a paediatric problem since most adults with regular exposure to livestock become immune to the disease while children do not acquire such immunity [43]. Exposure could occur through inhalation of aerosolized *Brucella* organism or direct consumption of contaminated raw meat or offals which are common in Nigerian abattoirs [22].

For this study, two serological tests were used to check seropositivity of cattle to brucellosis using serum samples. The RBT gave a seropositivity of 7.8% (97/1241), 77.3% (75/97) of which were further supported by cELISA. This is consistent with results from previous studies in south-western Nigeria [26, 27, 44, 45] which indicate moderate level of the disease. The seropositivity recorded in this study could be attributed primarily to lack of policy on brucellosis control in Nigeria [26]. Notably, since there is no standard ante-mortem inspection, many sick and moribund animals, mostly old females, are slaughtered at this abattoir, invariably contributing to the moderately high location prevalence observed. More so, another reason for the high seropositivity recorded could be attributed to the source of animals. Cattle sourced at Bodija Abattoir are from brucellosis endemic region of northern Nigeria [12, 46, 47] and parts of Africa [48, 49]. This problem is further accentuated by unrestricted trans-border animal movement of infected cattle which are sold at different cattle markets in Nigeria [50]. Again, our findings reveal that cows were almost three times more likely to be seropositive to *Brucella* spp than bulls. This is consistent with other studies [51–54]. According to Kebede et al. (2008) [55], bulls are usually kept for relatively shorter duration in breeding herds than the cows and thus, lowering the exposure of males when compared to females. Furthermore, cows are not sold for slaughter by herdsmen unless they are not doing well and the parameters of “not doing well” coincides with poor reproductive performance [56]. Importantly, the stress associated with pregnancy and calving tends to lower immunity of female animals [12] and this might also explain the observed difference. Importantly, the disease was only recorded among older sexually mature animals while the younger sexually immature ones were without infection. This result is similar to other findings [57,58] that recorded an analogous pattern of age distribution of disease. Thus affirming brucellosis as a disease of sexually mature animals, which is in tandem with earlier reports [12, 54, 59] where seropositivity to antibodies to *Brucella* was found to be higher in older animals. In addition, the longer presence of the older animals increases their chance of being exposed to Brucella infection [55]. Coupled with this, is the fact that animals become increasingly susceptible to *Brucella* infection as they approach sexual maturity [60, 61].

Major limitation of this study, is that it did not include bacteriological isolation and direct screening of the livestock workers as these would have provided better epidemiological insights to the work [62,63].

## Conclusion

Overall, lack of awareness about the zoonotic implications of brucellosis is a key finding among abattoir workers in this study. This is also reflected in their poor attitude and the need to use PPE. However, barriers to usage of PPE by the abattoir workers such as perceived inconvenience could be removed via organised education and awareness programmes as well as provision of free PPE to serve as an inducement and sanctioning of defaulters to serve as deterrent. In addition, since most of the workers engaged in risky health behaviour, it is imperative to encourage them to take appropriate actions needed to modify their risky behaviour towards a more favourable one. This information can be used to design intervention programmes targeting solutions to identified barriers to the prevention of brucellosis. Importantly, the roles of media, regulatory bodies/association leaders in enhancing cues to cognitive action remain invaluable in this regard. Importantly, our findings reiterate that brucellosis remains a major disease burden among trade cattle slaughtered in south-western Nigeria. Therefore, surveillance should be encouraged in order to limit the spread of disease, considering its public health importance. Finally, to promote public health, effective control of brucellosis in Nigeria can be achieved through; first, the control of the disease in cattle; second, adequate knowledge of the disease among humans; and third, modifying health behaviours especially among individuals at high risk of exposure, particularly the abattoir workers.

### What is known about this topic

- Brucellosis is a neglected zoonosis of global public health importance;
- Brucellosis affects a wide range of animals and can also affect humans leading to reproductive and diverse clinico-pathological sequelae;
- Serological methods have proven useful in the epidemiology of brucellosis in developing countries; however, isolation of the causal agent still remains the most dependable method of diagnosis.

### What this study adds

- This study found a moderate seroprevalence of 7.8% of brucellosis among cattle slaughtered in a major abattoir, Ibadan, southwestern Nigeria;
- Through daily observations on the use of personal protective equipment (PPE) and administration of questionnaire, we observed that the abattoir workers lacked awareness about zoonotic implications of brucellosis as well as the need to use PPE;
- Our findings show that barriers to the use of PPE by the workers; such as perceived inconvenience, could be removed via organised educational and awareness programmes; also, the provision of free PPE (particularly gloves) could serve as an inducement, while sanction of defaulters will be a deterrent to promote protection against brucellosis among abattoir workers.

## Competing interests

The authors declare no competing interest.
